# Application of Physiologically Based Pharmacokinetic Modeling to Predict Drug–Drug Interactions between Elexacaftor/Tezacaftor/Ivacaftor and Tacrolimus in Lung Transplant Recipients

**DOI:** 10.3390/pharmaceutics15051438

**Published:** 2023-05-08

**Authors:** Eunjin Hong, Eugeniu Carmanov, Alan Shi, Peter S. Chung, Adupa P. Rao, Kevin Forrester, Paul M. Beringer

**Affiliations:** 1 Department of Clinical Pharmacy, Mann School of Pharmacy and Pharmaceutical Sciences, University of Southern California, 1985 Zonal Ave, Los Angeles, CA 90033, USA; eunjinho@usc.edu (E.H.); carmanov@usc.edu (E.C.); alanshi@usc.edu (A.S.); kforrest@usc.edu (K.F.); 2 Division of Pulmonary and Critical Care Medicine, Department of Medicine, Keck School of Medicine, University of Southern California, 1975 Zonal Ave, Los Angeles, CA 90033, USA; peter.chung@med.usc.edu (P.S.C.); purush.rao@med.usc.edu (A.P.R.); 3 USC Anton Yelchin CF Clinic, 1510 San Pablo St, Los Angeles, CA 90033, USA

**Keywords:** physiologically based pharmacokinetic (PBPK), cystic fibrosis transmembrane conductance regulator (CFTR) modulator therapy, lung transplant, drug-drug interaction (DDI), intestinal cytochrome P450 3A4

## Abstract

Elexacaftor/tezacaftor/ivacaftor (ETI) treatment has potential benefits in lung transplant recipients, including improvements in extrapulmonary manifestations, such as gastrointestinal and sinus disease; however, ivacaftor is an inhibitor of cytochrome P450 3A (CYP3A) and may, therefore, pose a risk for elevated systemic exposure to tacrolimus. The aim of this investigation is to determine the impact of ETI on tacrolimus exposure and devise an appropriate dosing regimen to manage the risk of this drug–drug interaction (DDI). The CYP3A-mediated DDI of ivacaftor–tacrolimus was evaluated using a physiologically based pharmacokinetic (PBPK) modeling approach, incorporating CYP3A4 inhibition parameters of ivacaftor and in vitro enzyme kinetic parameters of tacrolimus. To further support the findings in PBPK modeling, we present a case series of lung transplant patients who received both ETI and tacrolimus. We predicted a 2.36-fold increase in tacrolimus exposure when co-administered with ivacaftor, which would require a 50% dose reduction of tacrolimus upon initiation of ETI treatment to avoid the risk of elevated systemic exposure. Clinical cases (N = 13) indicate a median 32% (IQR: −14.30, 63.80) increase in the dose-normalized tacrolimus trough level (trough concentration/weight-normalized daily dose) after starting ETI. These results indicate that the concomitant administration of tacrolimus and ETI may lead to a clinically significant DDI, requiring the dose adjustment of tacrolimus.

## 1. Introduction

The introduction of the Cystic Fibrosis Transmembrane Conductance Regular (CFTR) modulator, a triple combination of elexacaftor, tezacaftor, and ivacaftor (ETI, TRIKAFTA^®^), has led to substantial improvements in lung function for people with cystic fibrosis (pwCF) [1]. Treatment with ETI has demonstrated a significant increase in forced expiratory volume in one second (FEV_1_) and a decrease in pulmonary exacerbations [2,3]. In addition, ETI therapy has potential benefits for CF lung transplant recipients, as it can mitigate non-pulmonary manifestations of CF, such as nutritional deficiencies, sinus disease, and glucose metabolism abnormalities [4]. Furthermore, ETI has been reported to rapidly reduce the CF pathogen density in sputum, and ivacaftor specifically contributes to the enhanced antibiotic responsiveness of *Pseudomonas aeruginosa* [5,6].

In the case of lung transplant recipients, the use of ETI is still being explored, but its use requires careful attention to drug–drug interactions (DDI) with tacrolimus, a first-line immunosuppressive agent for organ transplant recipients. As tacrolimus is extensively metabolized by cytochrome P450 3A (CYP3A) present in both the intestine and liver, the inhibition of CYP3A by ivacaftor presents a potential risk for elevated systemic exposure to tacrolimus [7]. Inhibition of intestinal CYP3A can increase the bioavailability, while inhibition of hepatic CYP3A will reduce the clearance, both leading to increased drug exposure. The oral bioavailability of tacrolimus (<20%) is less than midazolam (31–72%), which suggests that tacrolimus may be more susceptible to intestinal metabolism than midazolam [8]. Maintaining therapeutic concentrations of tacrolimus is crucial to prevent rejection or adverse effects, as it has a narrow therapeutic index (trough concentrations of 10 to 15 ng/mL at months 1–3, 8–12 ng/mL at months 4–12) [7]. A reduced level of tacrolimus concentration may lead to the rejection of the organ. Alternatively, elevated concentrations of tacrolimus can contribute to an increased risk for adverse events, including excessive immunosuppression, which may predispose patients to infection, nephrotoxicity, and neurotoxicity [9]. Therefore, tacrolimus dose adjustment may be necessary in CF lung transplant recipients who receive ETI to prevent potential adverse drug reactions. While the product information for ivacaftor recommends safety monitoring for concomitant CYP3A substrate drugs, including tacrolimus, the extent of the DDI or appropriate dosing guidelines to correct the DDI remain unknown [10].

The aim of this study was to investigate the impact of ETI on tacrolimus exposure and determine appropriate dose adjustments of tacrolimus to overcome the interaction. We focused on the impact of ivacaftor on the CYP3A-mediated metabolism of tacrolimus, as elexacaftor and tezacaftor appear to have no significant effect on the activity of CYP3A [11]. The CYP-mediated drug interactions were evaluated using a physiologically based pharmacokinetic (PBPK) modeling approach. The predictive performance of PBPK for CYP enzyme-mediated metabolic DDIs has been well established [12]. Furthermore, many PBPK simulations have been submitted to regulatory agencies and accepted as the basis for dose adjustment on drug labels [13,14,15]. Our previous study quantitatively demonstrated the contribution of CYP3A to the metabolism of ETI, as well as the effect of CYP3A modulators on the pharmacokinetics of ETI based on PBPK modeling [16,17]. In the current work, we performed CYP3A inhibition assays using a luminescent probe, and incorporated the derived parameters into the ivacaftor model to predict the extent of CYP3A inhibition and its effect on the pharmacokinetics of CYP3A substrate drugs.

To further support the findings of the PBPK modeling, we report our experience with the combination of ETI and tacrolimus in a case series of lung transplant recipients. We hypothesized an increase in the dose-normalized tacrolimus trough levels would occur after the start of ETI.

The present study contributes towards improved treatment for CF lung transplant patients by providing guidance on the anticipated dose adjustments of tacrolimus upon initiation of ETI treatment. Furthermore, we present an optimized PBPK model of ivacaftor incorporating CYP3A4 inhibition potential, providing more insights into the CYP3A4-mediated interactions of ivacaftor as a perpetrator.

## 2. Methods

### 2.1. Development of PBPK Model

#### 2.1.1. Population Model

The PBPK models were implemented within the Simcyp Simulator (version 21; Certara, Sheffield, UK). To mimic the CF population, the distribution of ages and proportion of females were corrected to reflect the demographics of the CF population in the default healthy population library file (Sim-Healthy volunteers) provided in Simcyp^®^ [18]. Specifically, the frequency of the population aged 18–21 years was adjusted from 4.5% in the healthy population to 13.1% in CF. Further, the proportion of females was adjusted from 0.32 in the healthy population to 0.48 in CF. For the trial design, we used a population size of 100 (10 trials with 10 subjects in each trial).

#### 2.1.2. Ivacaftor Model

**Determination of CYP3A4 inhibition potential with P450 Glo luminescence assay**

Cryopreserved differentiated HepaRG cells were thawed and plated onto a flat 96-well plate at 72,000 cells/well. After 72 h of incubation, the CYP3A4 inhibition assay was performed following the manufacturer protocol with luciferin IPA, a selective CYP3A4 substrate that generates a luminescent signal upon metabolism [19]. The inhibition potential of ivacaftor was tested over a concentration range (1, 3, 10, 30, 100, 250 μM) inclusive of the clinically achieved plasma concentrations. Ketoconazole (0.1, 0.3, 0.1, 0.3, 1, 3, 10 μM) was used as a positive control. Each compound was added in triplicate and incubated with luciferin IPA for 120 min, followed by the addition of the luciferin detection reagent. Luminescence was measured using the BioTek Synergy H1 plate reader (Agilent Technologies, Santa Clara, CA, USA). Based on the competitive inhibition of ivacaftor and ketoconazole, the Ki values were calculated using the Cheng–Prusoff equation: Ki = IC50/(1 + [S]/Km), where [S] is the substrate concentration, and Km is the Michaelis constant. The [S] and Km were both 3 μM in our inhibition assay.

**Ivacaftor model development**

We previously validated a PBPK model of ivacaftor to quantify the interaction of perpetrator drugs in the CYP3A-mediated metabolism of ivacaftor [16]. Briefly, the ivacaftor model consists of the advanced dissolution, absorption, and metabolism (ADAM) model with minimal PBPK. Ivacaftor is a lipophilic compound with a logP of 5.68, and it binds avidly to albumin, with a fraction of unbound drug in plasma (fu) of 0.001. In vitro studies and clinical DDI data suggest that ivacaftor is predominantly eliminated through CYP3A4-mediated hepatic metabolism [11]. The fraction of ivacaftor being metabolized by CYP3A4 (fmCYP3A4) was set to 98% to capture the observed drug interactions of ivacaftor with the strong CYP3A4 modulators, ketoconazole or rifampin. In the present work, we additionally incorporated the CYP3A4 inhibition potential of ivacaftor obtained from the in vitro luminescence assay into the model to assess the potential DDI with CY3A4 substrate drugs. The unbound fraction in the assay incubation was assumed to be the same as the unbound fraction in plasma, based on the findings of Page et al. [20]. Alternatively, the utilization of fu^0.5^ as the unbound fraction in the incubation was not employed due to its reduced predictive capacity for the observed interactions of ivacaftor with midazolam.

#### 2.1.3. Tacrolimus Model Development

To our knowledge, there are no existing tacrolimus PBPK models verified for quantitation of the DDI with CYP3A modulators. Therefore, we developed the model of tacrolimus by incorporating drug parameters from literature [21,22,23,24] and tacrolimus model parameters published by Emoto et al. [25] to recapitulate the observed DDIs of tacrolimus (Table 1).

The tacrolimus model consists of first-order oral absorption and a minimal PBPK model. The drug-specific physicochemical properties, such as Log P_o:w_, protein binding, and blood-to-plasma ratio, as well as Caco-2 cell permeability, which is involved in drug absorption, were collected from Gertz et al. [21,22]. The *P*_eff,man_ was predicted to be 3.52 by the Simcyp calculator using these parameters. The absorption parameters, including *f*_a_, *k*_a_, lag time, and distribution parameters, including *k*_in_, *k*_out_, *V*_sac_, were obtained from a previously published model of tacrolimus by Emoto et al. [25]. For the elimination parameters, the enzyme kinetic model was used to simulate the CYP3A-derived metabolism of tacrolimus. The Km and Vmax involved in CYP3A metabolism were obtained from in vitro microsomal studies conducted by Dai et al. [23]. As tacrolimus undergoes minimal urinary excretion, with less than 1% of the dose being excreted unchanged in urine, the renal clearance was set to 0 L/h [24]. We did not incorporate the P-glycoprotein (P-gp) transport in the model. Although several studies have reported tacrolimus as a substrate of P-gp, its effect on tacrolimus absorption has been inconsistent [26,27,28]. Further, the unchanged tacrolimus concentration accounted for less than 1% of the total radioactivity excreted in feces, suggesting that hepatic P-gp has a minimal effect on tacrolimus PK.

### 2.2. PBPK Model Verification

#### 2.2.1. Plasma or Blood Pharmacokinetic Simulations

The plasma PK profile of ivacaftor following multiple-dose administration of 150 mg twice daily was simulated to verify the performance of the PBPK models after incorporating the CYP3A4 inhibition potential. Ivacaftor was orally administered under fed conditions to mimic the clinical setting, where a fat-containing food is required for optimal absorption. The simulated data were qualified using the plasma PK data observed in an adult CF population [11].

The whole-blood PK profiles of tacrolimus following single-dose administration (0.05 mg/kg oral or 0.01 mg/kg iv infusion for 4 h) were simulated to verify the performance of the tacrolimus model. Tacrolimus was administered under fasted conditions to mimic the clinical study. The simulated data were qualified using the blood PK data observed in a healthy adult population [24].

The prediction accuracies for the area under the curve (AUC) and maximum plasma concentration (C_max_) values were calculated as a ratio of mean observed over mean predicted values. Successful model performance was defined a priori by ratios of AUC and C_max_ within a twofold range, as previously described [29,30].

#### 2.2.2. DDI Simulations

Upon accurate recapitulation of the pharmacokinetics, the models were further evaluated using the clinical DDI data to confirm their suitability for assessing the DDI liability of a victim or perpetrator. For verification simulations, the dose and schedule of drugs were matched to the design of the corresponding clinical DDI study performed in healthy volunteers.

The validity of the ivacaftor CYP3A perpetrator DDI model was established by comparing the simulated DDIs with those observed in clinical studies with midazolam or ethinylestradiol, which are sensitive or weak CYP3A substrates, respectively [31]. The input parameters of these CYP3A substrates are available in the compound library of Simcyp version 21: SV-Ethinylestradiol and Sim-Midazolam. For ivacaftor–midazolam, ivacaftor 150 mg was administered orally twice daily for 6 days, and a single oral dose of midazolam 2 mg was administered on day 6. For the ivacaftor–ethinylestradiol situation, ivacaftor 150 mg was administered orally twice daily for 28 days, while ethinylestradiol oral 0.035 mg was administered once daily for 21 days.

The validity of the tacrolimus CYP3A victim DDI model was established by comparing the simulated DDIs with those observed in clinical studies with posaconazole or voriconazole, which are strong CYP3A inhibitors [7]. The PBPK models for posaconazole and voriconazole as CYP3A inhibitors were built according to the publications by Hong and Li et al., respectively [32,33]. For the posaconazole–tacrolimus DDI study, posaconazole was administered orally at a dose of 400 mg twice daily for 7 days, and a single oral dose of tacrolimus 0.05 mg/kg was administered on day 7. For the voriconazole–tacrolimus DDI, voriconazole was administered orally at a dose of 400 mg twice daily for the first day, followed by 200 mg twice daily for the next 6 days, and a single oral dose of tacrolimus 0.1 mg/kg was administered on day 7.

To quantify the DDIs, the geometric mean ratios of AUC or C_max_ in the presence or absence of CYP3A modulators were determined. The assessment of the DDI prediction success was based on whether predictions fell within a twofold range of the observed data.

### 2.3. Model Application

#### 2.3.1. DDI Predictions of Tacrolimus with Ivacaftor

The verified PBPK models were applied to (1) predict the effect of ivacaftor on the PK of tacrolimus and (2) determine a potential dose alteration of tacrolimus to overcome the CYP3A inhibition mediated by ivacaftor. We first simulated the steady-state PK of tacrolimus alone at a dose of 0.02 mg/kg. Subsequently, we simulated the PK of tacrolimus when co-administered with ivacaftor 150 mg twice daily until a steady state was achieved. Additionally, we simulated adjusted dosing regimens of tacrolimus when co-administered with ivacaftor to determine the optimal regimen that could provide PK profiles bioequivalent to tacrolimus alone.

#### 2.3.2. Sensitivity Analysis of Tacrolimus–Ivacaftor DDI

Given the significant variability observed in the tacrolimus–ivacaftor DDI, we performed a mechanistic assessment of potential key factors contributing to this variability through sensitivity analysis. The sensitivity analyses were performed focusing on hepatic and intestinal CYP3A4 abundances, hematocrit, and serum albumin concentration to explore their potential impact on the DDI. The sensitivity to these parameters were assessed within a twofold range of the default setting of parameters in the healthy population.

### 2.4. Clinical Case Series

This was an IRB-approved, retrospective, single-center cohort study that reviewed all CF lung transplant recipients prescribed ETI post lung transplantation. Patients were included if they were 18 years of age or older with CF, with at least one *F508del* mutation/minimal function genotype or a mutation in the CFTR gene that is responsive to ETI based on in vitro data. To evaluate the impact of ETI on tacrolimus exposure, tacrolimus trough levels were recorded before and up to one month after starting ETI. Patients had to be clinically stable, with no significant changes in health status, or initiating new medications known to modulate CYP3A4 and/or P-gp one month before or after starting ETI. Patients lost to follow-up or with missing tacrolimus trough levels were excluded.

Collected data included age, sex, weight, baseline lung function, body mass index, CFTR genotype, additional comorbidities, transplant and ETI start dates, ETI and tacrolimus dosing regimens, concurrent CYP3A4 modulators, and tacrolimus blood levels.

Baseline lung function was defined as the highest forced expiratory volume in one second (FEV_1_) during the year prior to initiating ETI therapy, obtained from routine pulmonary function tests [34]. Percent predicted FEV_1_ (ppFEV_1_) was then extrapolated using the Global Lung Function Initiative 2012 prediction equation [35]. Tacrolimus troughs were measured through routine clinical laboratory blood draws and analyzed by validated immunoturbidometric protocols. To assess the effects of ETI, tacrolimus doses and concentrations within one month before and after ETI start were recorded. The steady state for tacrolimus and ETI were estimated to be attained within four to five half-lives after starting a regimen. Tacrolimus troughs were recorded at least 2–3 days after a dose change and 5–6.5 days post ETI initiation, based on elexacaftor’s longest half-life of the three CFTR modulators [36,37]. Furthermore, to explore potential variability in the adherence to therapy and the timing of blood draws, tacrolimus trough levels within one month post ETI were averaged. For two patients with a changed tacrolimus dose immediately prior to ETI initiation and without a corresponding trough level, we captured the next most recent dose and trough within a three-month period.

Differences in tacrolimus trough concentrations before versus after starting ETI were analyzed utilizing the Wilcoxon signed-rank test, using all patients with both pre- and post-ETI measurements, against the null hypothesis that the difference in tacrolimus trough concentrations would be zero or demonstrate no change. All statistical analyses were performed using GraphPad version 8.0.2, with a two-sided significance value of 0.05.

## 3. Results

### 3.1. Model Development and Verification

#### 3.1.1. Ivacaftor Inhibition Potential

**Figure 1:**
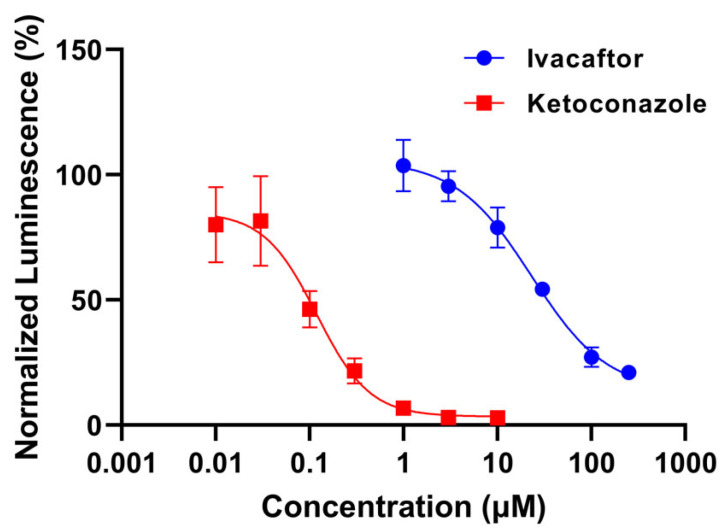
In vitro CYP3A4 inhibition by ivacaftor and ketoconazole at n = 3 per concentration. Both compounds demonstrated concentration-dependent inhibition of CYP3A4. Luminescence signal was normalized by dividing the luminescence of compound-treated well by the mean luminescence of DMSO-treated control wells and multiplying by 100.

Both the positive control inhibitor, ketoconazole, and the experimental compound, ivacaftor, showed concentration-dependent CYP3A4 inhibition, as evidenced by the decrease in normalized luminescence as the concentration of each test compound increased (Figure 1). However, ivacaftor demonstrated significantly weaker CYP3A4 inhibition effects, with an IC_50_ of 23.04 μM (95% CI: 15.22, 45.98) and K_i_ of 11.52 μM, when compared with ketoconazole, with an IC_50_ of 0.119 μM (95% CI: 0.073, 0.182) and K_i_ of 0.060 μM.

#### 3.1.2. PBPK Models of Ivacaftor and Tacrolimus Recapitulated Clinically Observed PK Profiles

Since the CYP3A inhibition potential of ivacaftor was added to the ivacaftor model, and ivacaftor is extensively metabolized by CYP3A, the model’s predictive performance was reassessed using observed plasma pharmacokinetic data from clinical trials [11]. The observed and simulated steady-state PK of ivacaftor following a standard dose administration of ivacaftor 150 mg q12h are summarized in Table 2. The predicted Cmax and AUC were 1.2 and 1.0 of the observed parameters, respectively.

The predictive performance of the tacrolimus model was also assessed using observed blood pharmacokinetic data upon a single dose of oral or intravenous administration [11]. In comparison with reported PK observations, the ratio of the predicted to observed parameters were in the range of 0.8–1.3 (Table 2).

#### 3.1.3. PBPK Models of Ivacaftor and Tacrolimus Recapitulated Observed DDI

Although the PK simulations verified the predicted PK of tacrolimus and ivacaftor, given that the PBPK models are intended to be applied for the characterization of DDIs involving CYP3A modulation, it is essential to verify the victim or perpetrator properties defined in the models by simulating independent clinical DDI studies with co-administered drugs. The model of ivacaftor recapitulated the observed DDIs with midazolam or ethinylestradiol (predicted AUC and Cmax ratio within the range of 0.91 to 1.09 of the observed values) (Table 3). The model of tacrolimus also recapitulated the observed DDIs with posaconazole or voriconazole (predicted AUC and Cmax ratio within the range of 0.98 to 1.20 of the observed values) (Table 4).

### 3.2. Model Application

#### 3.2.1. DDI Predictions of Tacrolimus with Ivacaftor

**Figure 2:**
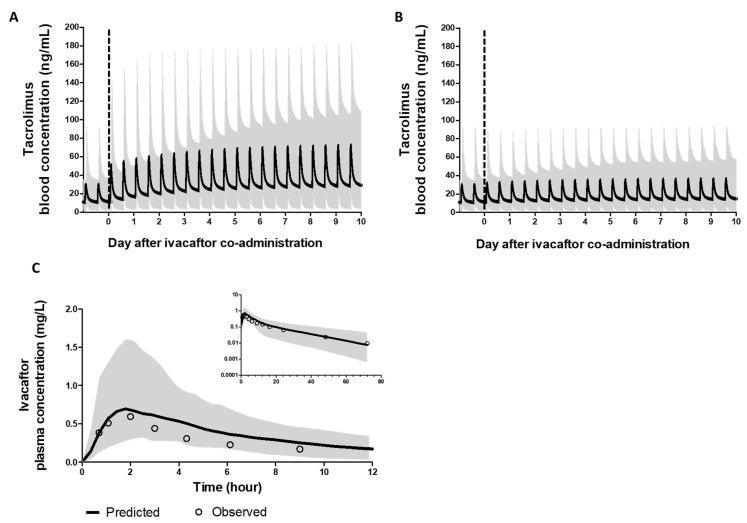
Blood concentration profile of tacrolimus and plasma concentration profile of ivacaftor, with the grey-colored area showing the range of predicted concentrations from 5th to 95th percentiles. (**A**) Continuing the same tacrolimus dose when ivacaftor was co-administered. (**B**) A 50% reduced tacrolimus dose when ivacaftor was co-administered. (**C**) Concentration profile of ivacaftor single dose (100 mg) administered.

The verified models were used to simulate the tacrolimus PK when co-administered with ivacaftor to determine the magnitude of the DDI. To mimic the clinical setting, we simulated the steady-state PK of tacrolimus, then added ivacaftor 150 mg twice daily while continuing the same tacrolimus dosing. The blood-concentration–time profile of tacrolimus when co-administered with ivacaftor is depicted in Figure 2A. The steady-state inhibition by ivacaftor is predicted to be established on day 6 of co-administration. The simulated geometric mean Cmax and AUC ratios of tacrolimus in the presence of ivacaftor at steady state are 2.28 (2.18, 2.39) and 2.36 (2.25, 2.47), respectively.

We next utilized the models to simulate the required dose adjustment of tacrolimus when co-administered with ivacaftor. Based on the simulated effect of ivacaftor, a 50% reduction in the tacrolimus dose was found to provide a steady-state PK profile within the bioequivalence limit of when tacrolimus was administered alone (Figure 2B). At the steady state, the AUC of the reduced dose was 112% of that predicted for tacrolimus alone. The reduced dose could be initiated on day 1 of ivacaftor co-administration, due to the competitive inhibition in CYP3A4, with the AUC of the reduced dose on day 1 being 105% of that of tacrolimus alone.

#### 3.2.2. Sensitivity Analysis

**Figure 3:**
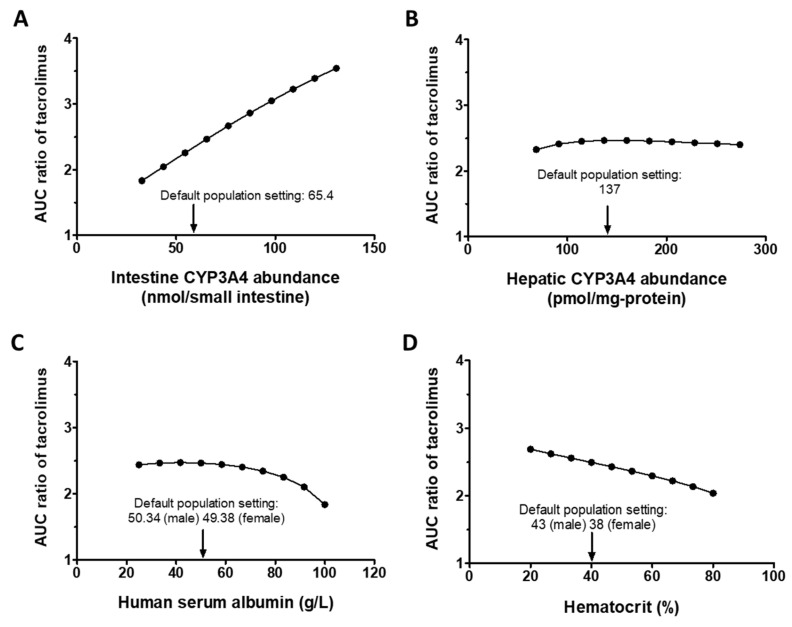
Sensitivity analysis results showing the effect of changes in physiological parameters on tacrolimus–ivacaftor DDI. Sensitivity analysis was conducted for (**A**) intestine CYP3A4 abundance, (**B**) hepatic CYP3A4 abundance, (**C**) human serum albumin, and (**D**) hematocrit. The 5 and 95 percentiles population values are (9.5, 121.3) for intestine CYP3A4 abundance, (44.6, 229.4) for hepatic CYP3A4 abundance, (40.4, 58.6) for human serum albumin, and (33, 47.6) for hematocrit.

First, the potential impact of hepatic and intestinal CYP3A4 abundances on DDIs between tacrolimus and ivacaftor were explored using sensitivity analysis. The simulated DDI was found to be more sensitive to intestinal CYP3A4 than hepatic CYP3A4 within the tested ranges for each factor (Figure 3A,B). The analysis demonstrated that a twofold decrease in intestinal CYP3A4 resulted in a 25.7% decrease in the AUC ratio, whereas a twofold increase in intestinal CYP3A4 led to a 43.8% increase in the AUC ratio.

The impact of hematocrit and serum albumin levels on the DDI of tacrolimus–ivacaftor were assessed within a twofold range of the default setting. The simulated DDI was found to be more sensitive to the hematocrit level compared with albumin concentrations, with minor changes in DDI predicted when the serum albumin level was varied in a range of 25–75 g/L (Figure 3C,D). Overall, the predicted DDI was most sensitive to changes in the intestinal CYP3A4 abundance and hematocrit, and not sensitive to the albumin level and hepatic CYP3A4 abundance.

### 3.3. Clinical Presentation

A total of 36 charts were reviewed for patients who received a lung transplant, and 23 were excluded due to not being prescribed ETI (N = 21), missing tacrolimus trough levels (N = 1), or lost to follow-up (N = 1). The demographics and clinical characteristics are summarized in Table 5. A full dose of ETI contained elexacaftor 200 mg once daily, tezacaftor 100 mg once daily, and ivacaftor 150 mg twice daily. Thirty one percent of the study population was on a reduced dose of ETI due to drug–drug interactions with potent CYP3A4 modulators, including itraconazole, posaconazole, voriconazole, and erythromycin. Reduced regimens included two orange tablets twice a week or alternating two orange and one blue tablet daily. These patients were further evaluated separately and compared to the rest of the cohort.

**Figure 4:**
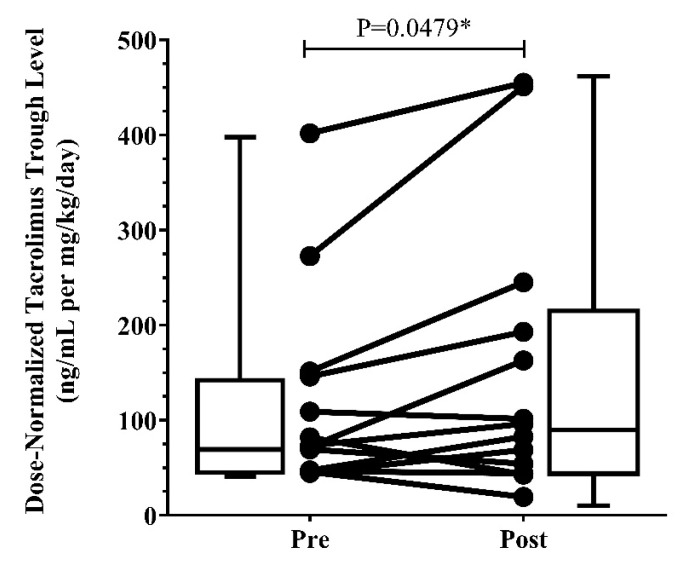
Dose-normalized tacrolimus trough levels pre- and post-ETI (N = 13). * *p*-value is less than 0.05.

The weight-normalized (WN) daily doses and dose-normalized (DN) trough levels of tacrolimus are summarized in Table 6. Co-administration of tacrolimus and ETI resulted in a statistically significant median 32% (IQR: −14.30, 63.80) increase in tacrolimus DN trough levels after starting ETI (*p* = 0.0479). Further subgroup analysis of 4 patients who previously were on CYP3A4 modulators and on a reduced ETI dose showed a more modest increase in tacrolimus DN trough levels of 22% (IQR:−32.64, 56.85). The WN daily dose before and after ETI was 0.096 (IQR: 0.044, 0.15) and 0.096 (IQR: 0.044, 0.14) mg/kg/day, respectively (*p* = 0.8911). Changes in DN tacrolimus trough levels per patient are illustrated in Figure 4.

## 4. Discussion

Clinical observations have demonstrated a clinically significant interaction between CYP3A4 inhibitors and tacrolimus [38]. The concomitant use of azole antifungal agents has been associated with the increased risk of adverse drug reactions of tacrolimus, including nephrotoxicity, due to the elevated systemic exposure of tacrolimus [39,40]. In addition, clarithromycin has been found to increase the tacrolimus concentration by fourfold, despite a 64% reduction in the tacrolimus dose upon the initiation of clarithromycin [41]. As ivacaftor is also a CYP3A4 inhibitor and requires long-term use, predicting CYP3A4-mediated drug interactions with tacrolimus and establishing appropriate dosing guidelines are of great importance. Our previous work has quantified the contribution of CYP3A4 to the metabolism of ETI via PBPK modeling [16]. In the present study, we determined the CYP3A4 inhibitory potency of ivacaftor and integrated the obtained parameters to establish an optimized PBPK model of ivacaftor to assess the potential CYP3A4 inhibition-mediated DDI with tacrolimus.

While the predicted AUC ratio for ethinylestradiol with co-administration of ivacaftor was 1.14, the predicted AUC ratios for midazolam and tacrolimus were 1.68 and 2.36, respectively, indicating more significant DDIs for sensitive CYP3A substrates. Based on the ivacaftor–tacrolimus DDI simulation, a twofold reduction in the tacrolimus dose is warranted. This finding is consistent with a case study by Doligalski et al. that reported a 50% decline in the tacrolimus dose following the initiation of ETI [42]. However, it is strongly recommended to perform therapeutic drug monitoring with this dose adjustment due to the variability of PK and the extent of the DDI of tacrolimus.

To support PBPK model findings, we further evaluated our CF lung transplant population. Clinical case reviews demonstrated a statistically significant increase of 32% (IQR: −14.30, 63.80) in DN tacrolimus trough levels, which is consistent with the results of our simulation. The observed WN daily dosing regimens before and after ETI initiation did not differ, with a possible explanation that the tacrolimus levels remained in the clinically desired range despite a potential increase, as suggested by the DN trough-level changes. We took measures to reduce potential sources of variability, such as capturing the average of tacrolimus troughs over the course of a month; however, due to the nature of a retrospective analysis, we could not eliminate all confounding variables. These include potential lack of medication adherence and trough timing, where the time of blood draw may not be representative of the true 12 h trough. Of note, the use of CYP3A4 modulators remained unchanged before and after the start of ETI in four of our patients; hence, they did not confound the effect of ETI on tacrolimus. However, when evaluating patients on reduced versus full doses of ETI, those on a reduced dose observed a more modest 22% (IQR: −32.46, 56.85) increase in DN tacrolimus troughs than those who were on a full dose, showing a 32% (IQR: −14.30, 67.94) increase. Thus, other CYP3A4 modulators could be masking the true effect of ETI on systemic exposure to tacrolimus. Changes in dietary habits with the start of ETI could be another confounding variable affecting tacrolimus trough levels. Tacrolimus is recommended to be taken with or without food, but consistently each time, to reduce potential variability in absorption [43]. However, ETI needs to be taken with a fatty meal for improved absorption, which could potentially alter the absorption of tacrolimus. The bioavailability of tacrolimus ranges from 5–93% and is significantly affected by the presence of food [36].

Limited literature exists describing tacrolimus dose/levels before and after the initiation of ETI, and the available data exhibit significant variability, which is compounded by disparities in the clinical information provided [44,45]. Doligalski et al. reported a 50% reduction in the dose requirement of tacrolimus, which was statistically significant with a similar size cohort as our study (N = 13) (39). In contrast, a smaller case series of nine post-lung-transplant recipients recently initiated on ETI, conducted by Benninger et al., did not demonstrate any significant differences in tacrolimus dosing requirements [46]. Ramos et al. examined a cohort of 30 patients across 14 CF Lung Transplant Consortium Transplant Centers and noted a dose increase for 7%, a dose decrease for 47%, and an unchanged dose for 38% of patients. Although we did not observe a change in the weight-normalized tacrolimus dosing regimen in our population, the rates of unnormalized dose changes paralleled those seen by Ramos et al. [47].

A large variability in DDI has been observed clinically, but a comprehensive assessment of the factors driving this variability is limited. In this study, the factors contributing to the DDI variability were assessed using sensitivity analysis. The findings highlight intestinal CYP3A4 abundance, which is highly variable in humans [48], as a major driving factor responsible for the individual variability seen in the DDI with ivacaftor. This indicates that gut metabolism is a critical factor in determining the extent of DDI, especially for the case of lung transplantation, where symptomatic gastroparesis is a frequent complication [49]. This result can be further supported by the comparison of midazolam and tacrolimus, which are both sensitive CYP3A substrates and exhibit a similar extent of DDIs with strong CYP3A inhibitors [50,51]. In a study involving co-administration with posaconazole 400 mg twice daily, the AUC ratios of midazolam and tacrolimus were 4.97 and 4.58, respectively [52]. However, in the present investigation of DDI with ivacaftor, which is a mild-to-moderate CYP3A inhibitor, the predicted AUC ratio of tacrolimus (2.36) was slightly higher than that of midazolam (1.68). This may be explained by the differences in the contribution of intestinal metabolism to these drugs. For weak CYP3A perpetrators, the extent of DDI mainly depends on intestinal interactions rather than hepatic interactions, as the drug concentration is higher in enterocytes than hepatocytes [53]. Tacrolimus appears to undergo more extensive intestinal metabolism when compared with midazolam, as suggested by the reported oral bioavailability of tacrolimus (<20%) compared to that of midazolam (31–72%) [8]. Furthermore, our PBPK simulations estimated that the fraction of drug escaping from intestinal metabolism was 0.30 and 0.72 for tacrolimus and midazolam, respectively, suggesting more extensive intestinal metabolism of tacrolimus than midazolam.

Given the significant contribution of intestinal metabolism in determining the degree of DDI, we further examined the impact of dose staggering on the DDI by administering the two drugs at different times. Our results indicate that a 3 h difference between doses caused a slight reduction in the extent of DDI (AUC ratio from 2.36 to 2.23), but it was not significant, suggesting that dose staggering has minimal effect on DDIs. It is likely that the prolonged Tmax (2–6 h) and long half-life of ivacaftor (15 h) enabled its gradual absorption and retention in the gut, thereby causing a lengthened duration of gut interaction.

CF patients have been found to exhibit a reduced serum albumin level by 15% when compared to healthy individuals [54]. Further, in lung transplant recipients, it has been reported that hematocrit levels may decrease to as low as 20% [55,56,57]. It should be noted that the incidence of anemia decreases over time after a transplant, with 52% of patients having anemia, with the majority of cases being mild after 3 years post-transplant, in contrast to the initial post-transplant period, where 98.2% of the population has anemia [57]. Therefore, these parameters were also included in the sensitivity analyses. While low albumin levels did not significantly affect DDI, low hematocrit levels led to higher DDI (AUC ratio of 2.69 at 20% of hematocrit), suggesting that more close monitoring may be required in this case due to the increased risk of elevated tacrolimus exposure. The sensitivity analysis provides mechanistic insights into factors contributing to the variability in tacrolimus DDI. More, this analysis can bridge the gap between the virtual and actual patient populations, thereby improving the predictability of a PBPK simulation.

A limitation of the study is that the simulation was based on a healthy population model, and population system parameters for CF lung transplant recipients were not incorporated due to the lack of data. This may introduce bias in the DDI predictions, as different pathophysiological conditions can alter a drug’s disposition. To address this limitation, we performed sensitivity analyses to assess the impact of varying the key physiological parameters on the model predictions. Additionally, we incorporated changes in the demography reflecting the CF population, leading to changes in the physiological parameters related to these covariates (e.g., liver weight). Previous studies have shown that differences in the PK of drugs in CF can be attributed to variations in body composition and plasma protein concentrations resulting from nutritional deficiencies [58]. However, recent improvements in CF care have resulted in an increase in the BMI, leading to a similar level to that of a normal healthy population [59]. Indeed, the PK of ETI has been reported to be similar in both healthy volunteers and CF patients in recent studies [11]. Further investigation is required to determine the impact of lung transplantation on the PK of ETI and tacrolimus. Another limitation is the use of the plasma unbound fraction (fu) as the unbound fraction in the assay incubation (fu,inc) for ivacaftor. We performed a sensitivity analysis by increasing fu,inc tenfold (0.001 to 0.01) to investigate the impact of varying fu,inc on the drug interactions with tacrolimus, and the predicted AUC ratios ranged from 2.36 to 1.80 within this range, which indicate a 50% dose reduction of tacrolimus. Regarding the clinical observations, the small sample size likely limits the power to detect a true effect of ETI on tacrolimus levels. The observed variability in the published data to date highlights the need for prospective clinical studies. The collection of data while controlling for medication adherence, timing of administration, and more rich sampling is needed to control confounding factors and reduce variability.

In conclusion, our study employed a PBPK modeling approach to demonstrate dose adjustment of tacrolimus is necessary when it is concomitantly administered with ETI. Since data demonstrating the health benefits of CFTR modulators have been increasing for CF lung transplant recipients, our findings offer valuable guidance for treatment strategies involving the combination of tacrolimus and ETI. Moreover, our study provides an established framework for modeling CYP3A-mediated drug interactions of ivacaftor and tacrolimus as perpetrator and victim drugs, respectively, which can be utilized to further investigate other clinically important drug interactions.

## Figures and Tables

**Table 1:** Parameters Used to Develop the Tacrolimus Model in Simcyp**^®^** Version 21 (Certara).

Parameter	Value	Source
Physiochemical properties		
Molecular weight (g/mol)	804.02	Drug label
Log P_o:w_	3.3	Gertz et al. [21]
Compound type	Neutral	Gertz et al. [21]
B/P	35	Gertz et al. [21]
fu_p_	0.013	Gertz et al. [21]
Absorption		
Absorption model	First-order model	
Caco-2 permeability (10^−6^ cm/s)	13.1	Gertz et al. [22]
Scalar	2.157	Gertz et al. [22]
fu_gut_	1	Default
*k*_a_ (h^−1^)	3.68	Emoto et al. [25]
*f*_a_	1.0	Emoto et al. [25]
Lag time (h)	0.43	Emoto et al. [25]
Qgut (L/h)	13.3	Predicted by Simcyp
*P*_eff,man_ (×10^−4^ cm/s)	3.52	Predicted by Simcyp
Distribution		
Distribution model	Minimal PBPK model	
*k*_in_ (h^−1^)	0.68	Emoto et al. [25]
*k*_out_ (h^−1^)	0.10	Emoto et al. [25]
*V*_sac_ (l/kg)	10.8	Emoto et al. [25]
*V*_ss_ (l/kg)	18.0	Predicted by Simcyp Method 1
Elimination		
**13-*O*-desmethylation**		
CYP3A4 Vmax (pmol/min/pmol CYP)	8	Dai et al. [23]
CYP3A4 Km (μM)	0.21	Dai et al. [23]
CYP3A5 Vmax (pmol/min/pmol CYP)	17	Dai et al. [23]
CYP3A5 Km (μM)	0.21	Dai et al. [23]
**12-hydroxylation**		
CYP3A4 Vmax (pmol/min/pmol CYP)	0.6	Dai et al. [23]
CYP3A4 Km (μM)	0.29	Dai et al. [23]
CYP3A5 Vmax (pmol/min/pmol CYP)	1.4	Dai et al. [23]
CYP3A5 Km (μM)	0.35	Dai et al. [23]
CYP3A4, 3A5 ISEF	0.24	Simcyp default
Renal clearance (L/h)	0	Moller et al. [24]

B/P, blood-to-plasma ratio; CL_int_, intrinsic clearance; CL_po_, in vivo oral clearance; *f*_a_, fraction available from dosage form; fu_gut_, fraction unbound in the enterocyte; fu_p_, fraction unbound in plasma; ISEF, inter-system extrapolation factor; *k*_a_, absorption rate constant; *k*_in_ and *k*_out_, first-order rate constants describing the drug transfer to a single adjusting compartment; Km, Michaelis constant; Log P_o:w_, logarithmic partition coefficient octonal:water; *P*_eff,man_, effective permeability in man; p*K*_a_, logarithm of acid dissociation constant; *Q*, inter-compartment clearance; Qgut, flow rate for overall delivery of drug to the gut; Vmax, maximum metabolic rate; *V*_sac_, single adjusted compartment volume; *V*_ss_, volume of distribution at steady state.

**Table 2:** Comparison of simulated and observed pharmacokinetic parameters for model verification.

PK Study		PK Parameters				
Drug	Regimen		Simulated		Observed	
			C_max_ (ng/mL)	AUC * (ng∙h/mL)	C_max_ (ng/mL)	AUC * (ng∙h/mL)
**ivacaftor**	**150 mg** **q12h** **oral**	Mean	1536	12,184	1270	12,100
		SD	1085	6641	353	4170
		Simulated/ observed	1.2	1.0		
**tacrolimus**	**0.05 mg/kg** **single dose** **oral**	Mean	37.5	398	37.8	307
		SD	28.1	320	16.0	251
		Simulated/ observed	1.0	1.3		
**tacrolimus**	**0.1 mg/kg** **single dose** **iv infusion** **(4 h)**	Mean	16.2	404	21.4	378
		SD	7.2	204	8.0	109
		Simulated/ observed	0.8	1.1		

* AUC (0–12 h) for ivacaftor, AUC (0–108 h) for tacrolimus oral, AUC (0–264 h) for tacrolimus iv infusion.

**Table 3:** Summary of the simulated vs. observed Geometric Mean Ratio (GMR) of PK parameters for co-administered drugs (victim drug) in the presence of ivacaftor (perpetrator drug).

Drug	PK Parameters	Simulated GMR (90% CI)	Observed * GMR (90% CI)	Ratio (Simulated/Observed)
Midazolam +/− ivacaftor	Cmax Ratio	1.51 (1.48, 1.55)	1.38 (1.26, 1.52)	1.09
	AUC Ratio	1.68 (1.64, 1.71)	1.54 (1.39, 1.69)	1.09
Ethinylestradiol +/− ivacaftor	Cmax Ratio	1.11 (1.10, 1.12)	1.22 (1.10, 1.36)	0.91
	AUC Ratio	1.14 (1.13, 1.15)	1.07 (1.00, 1.14)	1.07

* The observed GMR data were obtained from [31].

**Table 4:** Summary of the simulated vs. observed Geometric Mean Ratio (GMR) of PK parameters for tacrolimus (victim drug) in the presence of co-administered drugs (perpetrator drug).

Drug	PK Parameters	Simulated GMR (90% CI)	Observed * GMR (90% CI)	Ratio (Simulated/Observed)
tacrolimus +/− posaconazole	Cmax Ratio	2.4 (2.2, 2.5)	2.0 (2.0, 2.4)	1.20
	AUC Ratio	4.4 (4.0, 4.8)	4.5 (4.0, 5.2)	0.98
tacrolimus +/− voriconazole	Cmax Ratio	2.0 (1.9, 2.1)	2.0 (1.9, 2.5)	1.00
	AUC Ratio	3.2 (3,0, 3.4)	3.0 (2.7, 3.8)	1.07

* The observed GMR data were obtained from [7].

**Table 5:** Demographics and Clinical Characteristics of Patients.

Characteristic		Included Patients (N = 13)
Age		
	Median (25%, 75%)	37 (36.5, 52.5)
Female sex—no. (%)		6 (46)
Percentage of predicted FEV_1_		
	Median (25%, 75%)	92 (70.5, 104.5)
Body mass index		
	Median—kg/m^2^ (25%, 75%)	22.5 (20.6, 23.75)
CFTR Mutation—no. (%)		
	F508del/F508del	6 (46)
	F508del/minimal function	5 (38)
	F508del/residual function	1 (8)
	Other genotype	1 (8)
Comorbidities—no. (%)		
	Chronic rhinosinusitis	13 (100)
	Cystic fibrosis-related diabetes	8 (61)
	Gastrointestinal manifestations	7 (54)
	Two or more comorbidities	9 (69)
Duration since transplant and ETI start		
	Median—years (25%, 75%)	10.27 (4.19, 15.26)
ETI dose—no. (%)		
	Full	9 (69)
	Reduced	4 (31)
On other CYP3A4 modulators—no. (%)		4 (31)

**Table 6:** Patient tacrolimus dosing regimen pre and post initiation of ETI treatment.

	Pre-ETI			Post-ETI			
	TAC Dose	Laboratory Examinations		TAC Dose	Laboratory Examinations		
Patient Number	WN Daily Dose (mg/kg/day)	TAC Trough Concentration (ng/mL)	TAC DN Trough Level (ng/mL per mg/kg/day)	WN Daily Dose (mg/kg/day)	TAC Trough Concentration (ng/mL)	TAC DN Trough Level (ng/mL per mg/kg/day)	Percent Difference DN Trough (%)
1	0.178	8.40	47.25	0.171	14.10	82.64	74.90
2	0.028	7.70	272.58	0.022	9.75	451.10	65.49
3	0.051	7.50	146.00	0.050	9.73	193.04	32.22
4	0.165	11.40	69.29	0.156	8.45	54.29	−21.64
5	0.026	10.40	401.70	0.025	11.30	454.83	13.23
6	0.037	5.60	151.20	0.039	9.50	245.10	62.10
7	0.160	7.30	45.68	0.176	3.40	19.30	−57.76
8	0.097	7.10	73.41	0.093	8.90	96.12	30.93
9	0.061	6.60	108.97	0.060	6.10	101.40	−6.95
10	0.139	11.40	81.98	0.130	5.58	42.88	−47.69
11 *	0.118	NA	46.37	0.106	6.70	62.88	35.60
12 *	0.132	NA	44.69	0.132	9.05	68.78	53.90
13	0.096	6.90	72.22	0.096	15.70	162.86	125.51
Median	0.096	7.50	73.41	0.096	9.05	96.12	32.33
IQR (25%, 75%)	0.044, 0.15	6.90, 10.40	46.81, 148.6	0.044, 0.14	6.40, 10.53	58.29, 219.10	−14.30, 63.80

Abbreviations: WN, weight normalized; TAC, tacrolimus; DN, dose normalized; NA, not available. * No steady-state trough level available immediately prior to ETI initiation; trough levels from prior dose were used to determine the DN trough.

## Data Availability

The authors confirm that the data supporting the findings of this study are available within the article.
